# Metastatic status of sentinel lymph nodes in breast cancer determined with photoacoustic microscopy via dual-targeting nanoparticles

**DOI:** 10.1038/s41377-020-00399-0

**Published:** 2020-09-16

**Authors:** Yanfeng Dai, Xiang Yu, Jianshuang Wei, Fanxin Zeng, Yiran Li, Xiaoquan Yang, Qingming Luo, Zhihong Zhang

**Affiliations:** 1 Britton Chance Center and MOE Key Laboratory for Biomedical Photonics, School of Engineering Sciences, Wuhan National Laboratory for Optoelectronics–Huazhong University of Science and Technology, Wuhan, Hubei 430074 China; 2 School of Biomedical Engineering, Hainan University, Haikou, Hainan 570228 China

**Keywords:** Optical materials and structures, Optical techniques

## Abstract

Detection of sentinel lymph nodes (SLNs) is critical to guide the treatment of breast cancer. However, distinguishing metastatic SLNs from normal and inflamed lymph nodes (LNs) during surgical resection remains a challenge. Here, we report a CD44 and scavenger receptor class B1 dual-targeting hyaluronic acid nanoparticle (5K-HA-HPPS) loaded with the near-infra-red fluorescent dye DiR-BOA for SLN imaging in breast cancer. The small sized (~40 nm) self-assembled 5K-HA-HPPSs accumulated rapidly in the SLNs after intradermal injection. Compared with normal popliteal LNs (N-LN), there were ~3.2-fold and ~2.4-fold increases in fluorescence intensity in tumour metastatic SLNs (T-MLN) and inflamed LNs (Inf-LN), respectively, 6 h after nanoparticle inoculation. More importantly, photoacoustic microscopy (PAM) of 5K-HA-HPPS showed a significantly distinct distribution in T-MLN compared with N-LN and Inf-LN. Signals were mainly distributed at the centre of T-MLN but at the periphery of N-LN and Inf-LN. The ratio of PA intensity (R) at the centre of the LNs compared with that at the periphery was 5.93 ± 0.75 for T-MLNs of the 5K-HA-HPPS group, which was much higher than that for the Inf-LNs (*R* = 0.2 ± 0.07) and N-LNs (*R* = 0.45 ± 0.09). These results suggest that 5K-HA-HPPS injection combined with PAM provides a powerful tool for distinguishing metastatic SLNs from pLNs and inflamed LNs, thus guiding the removal of SLNs during breast cancer surgery.

## Breast cancer: nanoparticles highlight problematic lymph nodes

Fluorescent nanoparticles designed by researchers in China will help surgeons detect key sites where breast cancer spreads around the body. Tumours undergoing metastasis start by targeting sentinel lymph nodes, which are difficult to distinguish from normal or inflamed lymph nodes. Zhihong Zhang at Huazhong University of Science and Technology, Wuhan and co-workers, designed nanoparticles just 40 nm in size coated in hyaluronic acid, which binds to proteins overexpressed by breast cancer cells. The nanoparticles also include fluorescent dye molecules that are easily detected by photoacoustic microscopy. Six hours after injecting the nanoparticles into mice, the researchers observed strong photoacoustic signals at the centres of sentinel lymph nodes, while other lymph nodes showed signals only at their edges. This ability to distinguish important metastatic sites will guide surgeons in removing problematic tissue during breast cancer surgery.

## Introduction

Breast cancer is the most commonly diagnosed cancer and the leading cause of cancer-related mortality among women^1^. Globally, there were ~2.1 million newly diagnosed cases of breast cancer in 2018 alone, accounting for ~25% of cancer cases in women^2^. Despite advances in diagnosis and treatment, metastatic breast cancer, particularly hormone receptor-positive cancer, is considered incurable^3,4^. To balance the reduction in pain and benefits of an extended lifespan against the harm caused by treatment, a rational treatment programme should be based on tumour–node–metastasis staging^5^. In recent years, immunological surveillance of normal lymph nodes (LNs) has gradually enabled the replacement of complete axillary LN dissection by sentinel LN (SLN) biopsy (SLNB) as a means to accurately stage LNs^6^. Although SLNB in breast cancer has been indicated to be effective in reducing postoperative morbidity^7^, this procedure has some disadvantages. The standard SLNB method in a clinical setting involves injection of a technetium-labelled nanocolloid preoperatively into the breasts around the tumour, which increases patient concerns about further radiation exposure and reoperation^8^. Moreover, subsequent histological analysis of LN metastasis after SLNB is time-consuming and somewhat subjective. In addition, SLN dissection may cause complications such as lymphedema, shoulder dysfunction and numbness^9,10^. Therefore, the development of a new approach to accurately identify LN metastases intraoperatively will help surgeons choose appropriate treatment plans with improved effects and minimise the complications caused by unnecessary LN removal.

Several non-radiative approaches that can rapidly predict the metastatic status of SLNs have been developed. They include fluorescence imaging using a targeted fluorescent agent^11^, contrast-enhanced ultrasound using microbubbles^12^, nanoparticle-enhanced MRI^13,14^ and imaging by surface-enhanced Raman scattering^15^. Because these agents are mainly taken up by macrophages in LNs, these methods distinguish between tumours metastatic SLNs (T-MLN) and inflamed LNs (Inf-LN) according to the lower signal intensity of metastatic SLNs. However, metastatic LNs with a low signal intensity are less likely to be detected intraoperatively under a blurred visual field. Unfortunately, this drawback is further amplified when the lymphatic tissue is entirely occupied by tumour tissue at the late stage or when the afferent lymphatic vessels are blocked by metastatic tumours. Therefore, there is an urgent need to develop a new strategy based on the characteristics of the metastatic tumour itself to discriminate normal LNs (N-LN) and Inf-LN from SLNs in breast cancer. To selectively target breast cancer cells in SLNs, two factors must be taken into consideration. One is that the tracer agent should rapidly migrate to LNs by either a passive targeting mechanism, an active targeting mechanism, or both. The other factor is that the tracer agent should contain a specific ligand for the selective targeting of breast cancer cells.

Hyaluronic acid (HA), a linear mucopolysaccharide possessing a large number of negatively charged carboxyl groups, is an essential component of the extracellular matrix that is generated naturally in all living organisms^16^. Due to its outstanding biocompatibility and non-immunogenicity, HA and its derivatives have been clinically used as medical products for tissue repair and regeneration for several decades^17^. HA is also a well-known ligand of CD44, which is overexpressed in breast cancer^18^; thus, HA can serve as an ideal candidate for designing novel HA-based nanocarriers that selectively target breast cancer cells. However, the turnover of CD44 is modulated by occupancy with HA, which can easily lead to saturation^19^. In addition, glycosylation of CD44 negatively regulates its ability to recognise HA and inhibits CD44-mediated uptake efficiency^20^, suggesting that only HA-mediated CD44 targeting of tumour cells is insufficient to acquire images with a high signal-to-noise ratio. Therefore, a dual receptor-targeted drug delivery system will be helpful to target breast cancer cells more effectively.

**Fig. 1:**
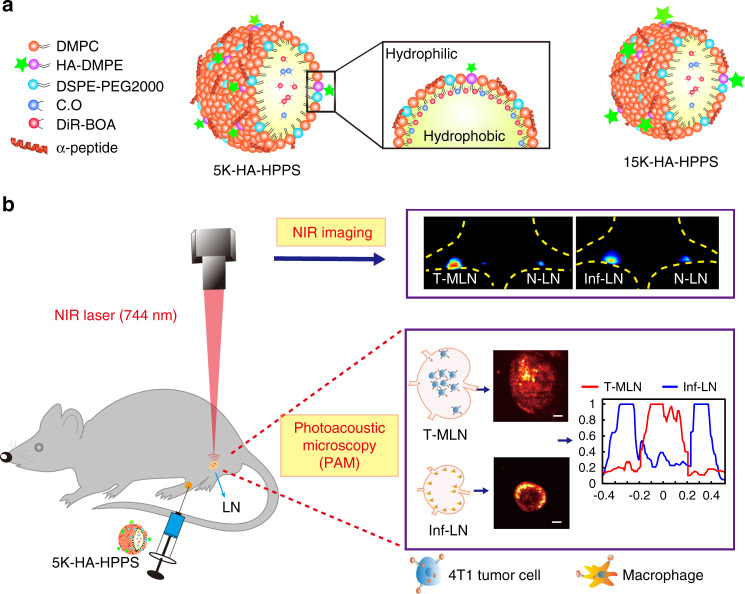
Design of dual-modality HA-HPPS nanoparticles for mapping sentinel lymph nodes in breast cancer. **a** Components and structures of the CD44 and SR-B1 dual-targeting HA-HPPS nanoparticles. **b** Dual-modality fluorescence and photoacoustic imaging of HA-HPPS in SLNs, which includes near-infra-red (NIR) fluorescence imaging for long-term monitoring of the accumulation and retention of HA-HPPS in SLNs and photoacoustic microscopy (PAM) for intraoperative determination of the metastatic status of SLNs in breast cancer

Particle size is the most important factor in nanoparticle migration to LNs. Although the optimal size range remains a matter of debate, it is generally considered that ultrasmall nanoparticles (10–50 nm) show rapid uptake and long retention times in the lymphatic system^21,22^. Moreover, it is favourable to transport negatively charged nanoparticles into the lymphatic system in the interstitial fluid due to the negative charge on glycosaminoglycans in the extracellular matrix^23^. Previously, we developed a high-density lipoprotein (HDL)-mimicking peptide–phospholipid scaffold (HPPS) with hydrodynamic diameters of 10–30 nm^24^ that can migrate to LNs by size-dependent passive transport^21,25^. Moreover, HPPS nanoparticles can target scavenger receptor class B type 1 (SR-B1), which is expressed on breast cancer cells^26^. Herein, we developed a novel dual-targeting nanoparticle by inserting the negatively charged HA-1,2-dimyristoyl phosphatidylethanolamine (DMPE) into a single-layer phospholipid of HPPS, denoted HA-HPPS. Importantly, this dual-targeting nanoparticle has a phospholipid monolayer shell containing DiR-BOA, a near-infra-red (NIR) fluorophore, which can be used for both fluorescence imaging and photoacoustic microscopy (PAM)^27,28^. We can continuously monitor changes in the DiR-BOA fluorescence signal of LNs in a mouse model (e.g. inflammatory or tumour model) by using a wide-field fluorescent imaging system that has the advantages of sensitivity, convenience and non-invasiveness. By providing deep penetration and high spatial resolution, PAM has great potential for the 3D visualisation of photoacoustic signal distribution in intact LNs. In this proof-of-concept study (Fig. 1), we hypothesised that HA–HPPS combined with fluorescence/photoacoustic imaging can enable the visualisation of SLNs in a rapid and prolonged manner through size- and charge-dependent passive and active targeting of both CD44 and SR-B1, which can guide the removal of SLNs from patients during breast cancer surgery.

## Results

### Synthesis and characteristics of the CD44 and SR-B1 dual-targeted lipid nanoparticles

**Fig. 2:**
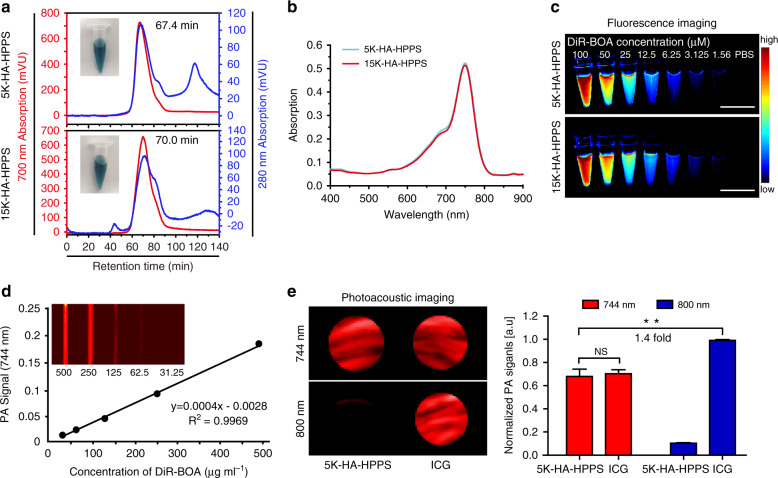
In vitro characteristics and optical properties of 5K-HA-HPPS and 15K-HA-HPPS. **a** FPLC profiles and photographs of 5K-HA-HPPS and 15K-HA-HPPS. **b** 5K-HA-HPPS and 15K-HA-HPPS absorption spectra. **c** In vitro fluorescence imaging of 5K-HA-HPPS and 15K-HA-HPPS with different DiR-BOA concentrations (μM): 100, 50, 25, 12.5, 6.25, 3.125 and 1.56. Scale bar: 1 cm. **d** In vitro PA imaging of 5K-HA-HPPS excited at 744 nm as a function of different concentrations. **e** The photoacoustic signal amplitude of PAM 5K-HA-HPPS and ICG at 744nm and 800 nm (left panel) and the quantitative analysis of 5K-HA-HPPS and ICG at 744 nm and 800 nm (right panel). Data are presented as the mean ± SD. **P < 0.01. *n* = 3

To synthesise CD44 and SR-B1 dual-targeted nanoparticles, we first synthesised 5K-HA-DMPE or 15K-HA-DMPE using 5-kDa HA- or 15-kDa HA-conjugated DMPE (Supplementary Fig. S1a). After purification, the chemical structures of HA, DMPE and HA-DMPE were determined by ^1^H NMR spectroscopy. The chemical shifts of the protons of the terminal methyl (0.9–1.0 ppm) and methylene (1.26 ppm) groups of DMPE and the N-acetyl group (1.93 ppm) of HA were used to determine HA-DMPE synthesis (Supplementary Fig. S1b). Then, HA-DMPE was inserted into a single-layer phospholipid of HPPS to form HA-HPPS, and the final product was purified using a fast protein liquid chromatography (FPLC) system. The FPLC profile, in which absorption curves at 280 nm and 700 nm represented HA-HPPS and DiR-BOA, respectively (Fig. 2a), showed that two main narrow peaks at retention times of 67.4 and 70 min represented 5K-HA-HPPS and 15K-HA-HPPS, respectively. Transmission electron microscopy (TEM) clearly showed that 5K-HA-HPPS and 15K-HA-HPPS had uniform spherical morphologies (Supplementary Fig. S2a). Dynamic light scattering (DLS) indicated that 5K-HA-HPPS and 15K-HA-HPPS were ~43 and ~38 nm in size, respectively (Supplementary Fig. S2b). Notably, these sizes were smaller than those of the 5K-HA emulsion (5K-HA-e, ~57.6 nm) and 15K-HA emulsion (15K-HA-e, ~53.6 nm) (Supplementary Fig. S2c). In addition, the zeta potential profiles indicated that 5K-HA-HPPS and 15K-HA-HPPS carried slight negative charges (−7.2 ± 0.6 and −5.6 ± 0.6 mV, respectively) (Supplementary Fig. S2d). The 5K-HA-HPPS and 15K-HA-HPPS absorption spectra both showed a maximum absorption peak at 748 nm (Fig. 2b). 5K-HA-HPPS and 15K-HA-HPPS fluorescence imaging were well correlated with the presented DiR-BOA concentrations (Fig. 2c). Next, we verified that the PA signals of the 5K-HA-HPPS solutions with different DiR-BOA concentrations were linearly dependent on the concentration (*R*^2^ = 0.9969) from 31.25 to 500 μg ml^−1^ (Fig. 2d). The PA signal of 5K-HA-HPPS was comparable to that of the FDA-approved NIR-PA contrast agent ICG at the same concentration at 744 nm, but the PA signal of ICG at 800 nm was much stronger than that of 5K-HA-HPPS and 1.4 times that of 5K-HA-HPPS at 744 nm (Fig. 2e). To verify the stability of HA-HPPS, the HA-HPPS core loaded with DiR-BOA was additionally labelled with fluorescein isothiocyanate (FITC) to form the dual-labelled FITC–HA-HPPS(DiR-BOA). Seminative SDS polyacrylamide gel electrophoresis (PAGE) indicated that both 5K-HA-HPPS and 15K-HA-HPPS were stable in 10% mouse plasma at 37 °C, even after continuous incubation for 24 h. As a control, 10% Triton-X 100 disrupted the structure of HA-HPPS, resulting in unrestricted diffusion of DiR-BOA fluorescence signals (Supplementary Fig. S2e). Finally, to assess the cytotoxicity of the nanoparticles, we examined the viability of RAW264.7 cells after incubation with different concentrations of HA-HPPS for 24 h using the (3-(4,5-dimethylthiazol-2-yl)-5-(3-carboxymethoxyphenyl)-2-(4-sulfophenyl)-2H-tetrazolium) (MTS) assay. As shown in Supplementary Fig. S2f, ~100% of the RAW264.7 cells survived with a wide range of HA-HPPS concentrations (peptide concentrations: 1–20 μM) after 24 h of incubation. These results suggested that the HA-based nanoparticles have good biocompatibility for in vivo applications.

### 5K-HA-HPPS migrates to LNs rapidly and efficiently

**Fig. 3:**
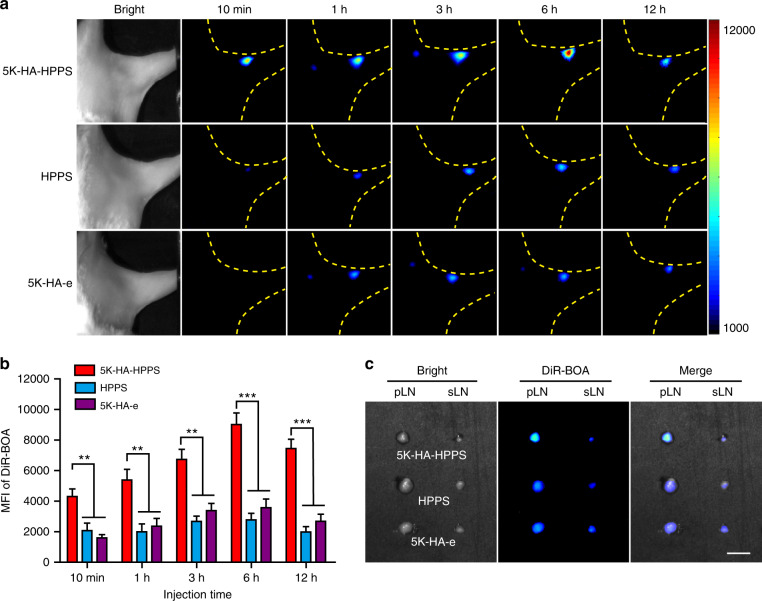
In vivo comparison of the migratory capabilities of 5K-HA-HPPS, HPPS and 5K-HA-e to pLNs. **a** Representative fluorescence images to observe the migratory capabilities of 5K-HA-HPPS, HPPS and 5K-HA-e to pLNs at 10min, 1, 3, 6 and 12 h after intradermal footpad injection. **b** Quantitative analysis of the mean fluorescence intensity of DiR-BOA in pLNs. **c** Fluorescence imaging was performed 12 h after injection on resected pLNs and sciatic LNs (sLNs). Scale bar: 2 mm. Data are presented as the mean ± SD (two-tailed *t*-test; *n* = 5 animals per group)

To explore the effects of HA-HPPSs of similar sizes and charges on the non-invasive imaging of LNs in vivo, we investigated the lymphatic trafficking ability of HA-HPPS in albino C57BL/6 mice. Following intradermal injection of HA-HPPS, HPPS, 5K-HA-e, or 15K-HA-e into the hind footpads of mice, wide-field fluorescence imaging was performed to record the migration and retention of the imaging tracers at different time points after different exposure times (30 s in Fig. 3a and 10 s in Supplementary Fig. S3). As shown in Fig. 3a, 5K-HA-HPPS migrated rapidly to popliteal LNs (pLNs), showing a strong fluorescence signal within 10 min. The mean fluorescence intensity (MFI) of DiR-BOA in pLNs gradually increased with time and reached a maximum at 6 h and was strong even at 12 h. However, pLNs injected with HPPS or 5K-HA-e presented only relatively weak fluorescence signals 1 h after injection. The MFIs of DiR-BOA in 5K-HA-HPPS were ~3.0- and ~2.3-fold greater than those of HPPS and 5K-HA-e at 6 h and ~3.2- and ~2.5-fold greater at 12 h, respectively (Fig. 3b). This result was confirmed by in vitro imaging of excised pLNs at 12 h (Fig. 3c). Interestingly, all sciatic LNs in the three groups showed similar signal intensities. These results suggested that 5K-HA-HPPS preferentially accumulated in pLNs and migrated to secondary LNs. When the exposure time was reduced to 10 s, clear fluorescence signals of DiR-BOA in the pLNs of the 5K-HA-HPPS group remained detectable at significantly stronger levels than those of the control groups (Supplementary Fig. S3). However, the fluorescence signals of DiR-BOA in the pLNs of the 15K-HA-HPPS group were very weak (Supplementary Fig. S4a). Moreover, there were no significant differences in the MFIs of DiR-BOA among the 15K-HA-HPPS, HPPS and 15K-HA-e groups (Supplementary Fig. S4b, c). These results indicated that 5K-HA-HPPS, rather than 15K-HA-HPPS, migrates rapidly and efficiently to LNs and can serve as a good LN imaging agent.

### Dual-targeting ability of 5K-HA-HPPS in 4T1 cells

**Fig. 4:**
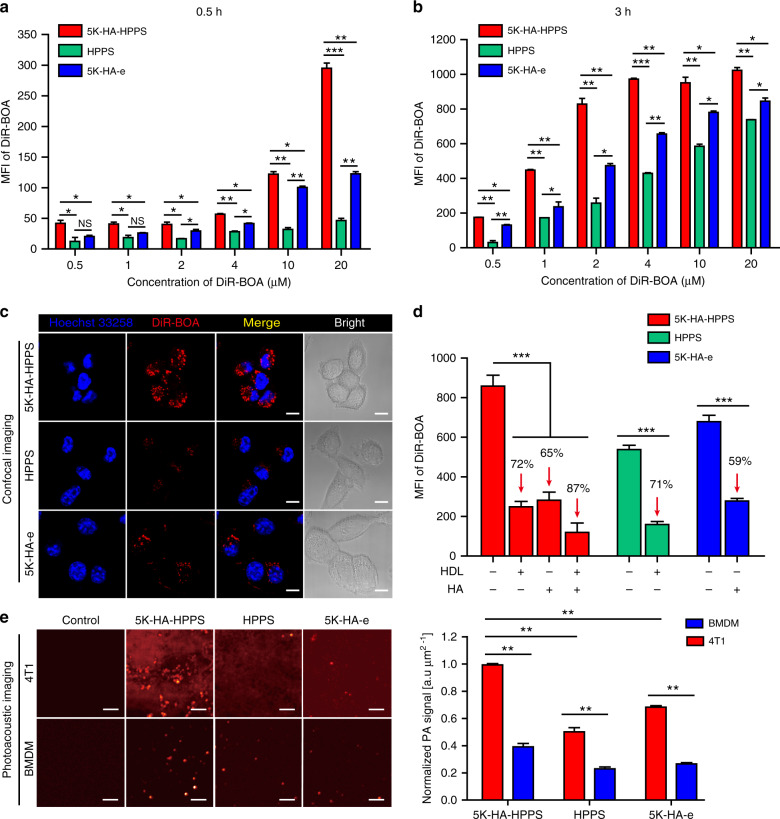
5K-HA-HPPS efficiently targeted 4T1 cells through CD44 and SR-B1 receptors in vitro. Flow cytometry was performed to analyse and compare the cellular uptake of 5K-HA-HPPS, HPPS and 5K-HA-e by 4T1 cells (5 × 10^4^ cells/well) in vitro after exposure to various concentrations for 0.5 h (**a**) and 3h (**b**). MFI: mean fluorescent intensity. **c** Confocal images for the nanoparticle-targeting ability to 4T1 cells (2 × 10^4^ cells/well). Scale bar: 50 μm. **d** Flow cytometry of 4T1 cells (5 × 10^4^ cells/well) pretreated for 3 h with free HDL protein and HA with 5K-HA-HPPS, HPPS and 5K-HA-e. The mass ratio of HDL to R4F in 5K-HA-HPPS and HPPS was 10:1. The mass ratio of free HA to HA in 5K-HA-HPPS and 5K-HA-e was 250:1. Blue: Hoechst 33258, red: DiR-BOA. **e** PAM and quantitative analysis of 4T1 cells and bone marrow-derived macrophages (BMDMs) incubated in vitro with 5K-HA-HPPS, HPPS and 5K-HA-e for 3 h. The spatial resolution of PAM was ~45 μm. Data are presented as the mean ± SD (two-tailed *t*-test; *n* = 3)

Having confirmed the ability of 5K-HA-HPPS to target LNs, we evaluated the ability of 5K-HA-HPPS to target 4T1 cells, which highly express SR-B1 and CD44, using flow cytometry (FCM) and confocal imaging. After 0.5 and 3 h of incubation, FCM showed that 4T1 cells took up nanoparticles in a concentration-dependent manner, and the MFI of DiR-BOA in 4T1 cells increased with incubation time (0.5–3 h) (Fig. 4a, b). The MFI of DiR-BOA in 4T1 cells treated with dual-targeted 5K-HA-HPPS was higher than that of single-targeted HPPS and 5K-HA-e, with a 6.3- and 2.4-fold difference, respectively, at a high concentration (20 μM DiR-BOA) after 0.5 h of incubation (Fig. 4a). Interestingly, at DiR-BOA concentrations of >2 μM (2–20 μM), the uptake of 5K-HA-e by 4T1 cells was stronger than that of HPPS (Fig. 4a, b). This effect may be due to the overexpression of CD44 on 4T1 cells and highly efficient uptake of 5K-HA-e. Moreover, after 3 h of incubation, confocal imaging demonstrated that the nanoparticles were distributed mainly in the cytoplasm, and the fluorescence intensity of 4T1 cells treated with 5K-HA-HPPS was significantly stronger than that of HPPS- and 5K-HA-e-treated cells (Fig. 4c). In addition, we detected the uptake of 5K-HA-HPPS by B16F10 melanoma cells, CT26 colon tumour cells and E0771 breast cancer cells, which also express CD44 and SR-B1. FCM showed that E0771 cells effectively took up 5K-HA-HPPS with a similar ability as 4T1 cells, and both B16 and CT26 cells also had a strong uptake of 5K-HA-HPPS (Supplementary Fig. S5). To further demonstrate that the endocytosis of nanoparticles by 4T1 cells is mediated by the CD44 and SR-B1 receptors, we designed a competitive inhibition experiment by treating the 4T1 cells with an excessive amount of free HDL protein and/or HA, which blocked the SR-B1 and CD44-mediated uptake of 5K-HA-HPPS, respectively. FCM showed that excessive HDL protein and HA dramatically inhibited the internalisation of HPPS (71% decrease) and 5K-HA-e (59% decrease) by 4T1 cells (Fig. 4d). In addition, when excessive HDL protein and HA were added simultaneously, the uptake of 5K-HA-HPPS decreased by 87% in 4T1 cells. Furthermore, we compared the uptake efficiency of 5K-HA-HPPS by 4T1 cells, bone marrow-derived macrophages (BMDMs) and RAW264.7 cells. The flow cytometry data showed that the efficiency of 5K-HA-HPPS uptake by 4T1 cells was much higher than that of BMDM cells, with a 3.5- and 1.8-fold difference at 2 μM and 10 μM DiR-BOA after 0.5 h of incubation and 3.9- and 2.8-fold after 3 h of incubation, respectively (Supplementary Fig. S6a). In addition, after 3 h of incubation, confocal imaging demonstrated that the fluorescence intensity of 4T1 cells treated with 5K-HA-HPPS was significantly stronger than that of BMDMs, RAW264.7 cells and bone marrow-derived dendritic cells (BMDCs) (Supplementary Fig. S6b). We used PAM to compare the uptake ability of 5K-HA-HPPS, HPPS and 5K-HA-e by 4T1 tumour cells and BMDMs after incubation for 3 h in vitro. As shown in Fig. 4e, the PAM signal in 4T1 cells was stronger than that in BMDM cells, and the PAM signal of 4T1 cells incubated with 5K-HA-HPPS was much stronger than that in the HPPS and 5K-HA-e groups. Collectively, these results indicated that dual-targeting of SR-B1- and CD44-mediated endocytosis can explain the increased cellular uptake efficiency of 5K-HA-HPPS by 4T1 cells.

**Fig. 5:**
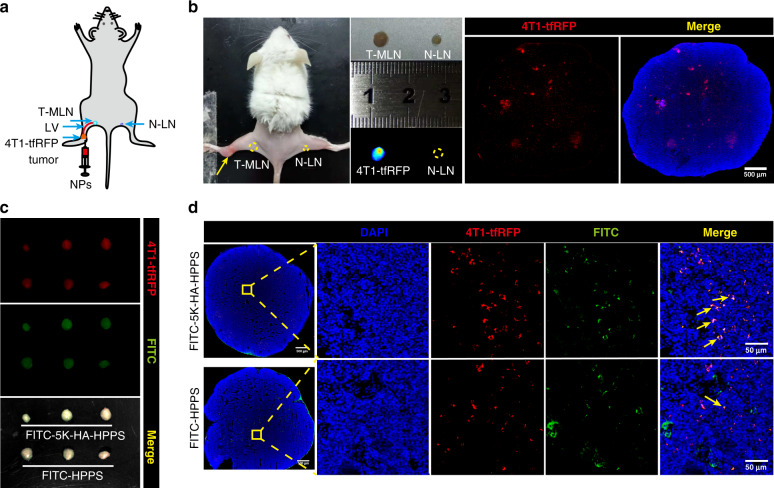
5K-HA-HPPS efficiently targeted 4T1 cells in vivo. **a** Schematic of LN tumour metastasis detection after intratumoural injection of NPs. **b** Successful establishment of the 4T1-tfRFP breast tumour LN metastasis model (left yellow dotted circle for T-MLN, right yellow dotted circle for N-LN), where the fluorescence signal of FITC–5K-HA-HPPS in the LNs shows metastasis of 4T1-tfRFP cells. Confocal imaging confirmed that 4T1-tfRFP tumour cells metastasised within pLNs. Scale bar: 500 μm. **c** Three weeks after hock inoculation of 4T1-tfRFP cells (5 × 10^5^ cells/mouse), wide-field fluorescence imaging of resected tumour-draining LNs (*n* = 3 mice/group) was performed at 6 h after intratumoural injection of FITC-labelled 5K-HA-HPPS and HPPS. Red: 4T1-tfRFP cells, green: FITC-labelled nanoparticles. *n* = 3 mice/group. **d** Confocal microscopy verified that FITC-labelled 5K-HA-HPPS colocalized with the majority of 4T1-tfRFP cells in tumour-draining LNs. Scale bar: 50 μm. Red: 4T1-tfRFP cells, blue: DAPI, green: FITC-labelled 5K-HA-HPPS

Next, the in vivo ability of 5K-HA-HPPS to target metastatic 4T1 cells was examined in a LN metastasis tumour model, which was established by hock inoculation of 4T1-tfRFP cells in the left legs of BALB/c mice (Fig. 5a). Three weeks after tumour inoculation, primary tumours had developed (Fig. 5b). Compared with N-LNs, T-MLNs were evidenced by their enlarged size and increased fluorescence intensity of the excised LNs (Fig. 5b). Confocal imaging of the T-MLN sections indicated that the 4T1-tfRFP cells were distributed mainly in the inner part of the LNs, whereas only a few tumour cells were distributed in the periphery (Fig. 5b). Then, FITC-labelled nanoparticles were injected into the primary tumours, and LNs were removed 6 h later. Overall, T-MLNs had bright fluorescence signals from both the tfRFP protein and FITC, indicating that the 4T1-tfRFP cells metastasised into the SLNs and that the nanoparticles accumulated in the SLNs (Fig. 5c). Confocal imaging of LN sections indicated that there were many 4T1-tfRFP cells inside tumour-draining LNs. More importantly, FITC-labelled 5K-HA-HPPS colocalized with the majority of the 4T1-tfRFP cells in SLNs, whereas tumour cells labelled with FITC–HPPS were rarely observed (Fig. 5d). Thus, 5K-HA-HPPS can effectively target 4T1 cells by dual-targeting CD44 and SR-B1 in vitro and in vivo.

### In vivo NIR fluorescence imaging for the detection of 4T1 cells in metastatic LNs

**Fig. 6:**
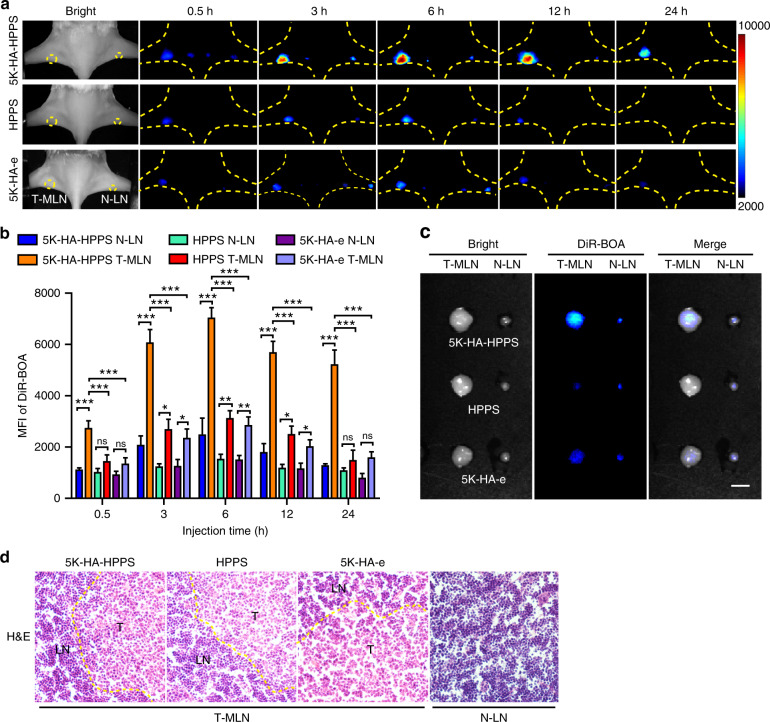
In vivo NIR fluorescence imaging of 5K-HA-HPPS for the detection of metastatic SLNs in breast cancer. **a** After hock inoculation of 4T1 tumour cells (5 × 10^5^ cells/mouse), 5K-HA-HPPS (upper), HPPS (middle) and 5K-HA-e (lower) were administered by intratumoural (left) or footpad injection (right), and NIR fluorescence imaging of pLNs was performed at 0.5, 3, 6, 12 and 24 h after injection. **b** Quantitative statistical analysis of the mean fluorescence intensity of DiR-BOA in pLNs. **c** Fluorescence images of excised LNs from BALB/c mice. Scale bar: 2 mm. **d** H&E staining of LNs from the tumour side and control side. Data are presented as the mean ± SD, *n* = 6 mice/group (two-tailed *t*-test)

Inspired by the fact that 5K-HA-HPPS was found to effectively target SLNs and metastatic 4T1 cells, we proposed that 5K-HA-HPPS can distinguish between metastatic and normal LNs. A LN metastasis model of 4T1 breast tumours was established as described above, but we did not use 4T1-tfRFP due to the slow growth of this cell line and the period required to establish the model. After injecting 5K-HA-HPPS, HPPS or 5K-HA-e into primary tumours and contralateral subcutaneous injection, wide-field fluorescence imaging was performed to record the migration and retention of imaging tracers at different timepoints. As shown in Fig. 6a, there was a dramatically higher tracer uptake in popliteal T-MLNs compared with contralateral N-LNs at all timepoints, and the DiR-BOA signal intensity from T-MLNs in the 5K-HA-HPPS group was significantly greater than the signal intensities in the HPPS and 5K-HA-e groups. Quantitative analysis indicated that the accumulation of 5K-HA-HPPS in T-MLNs was 3.3- and 4.25-fold higher than that in the contralateral N-LNs at 6 and 24 h, respectively (Fig. 6b). Moreover, the MFI of DiR-BOA in the T-MLNs of the 5K-HA-HPPS group was 2.16- and 2.45-fold higher than that in the HPPS and 5K-HA-e groups at 6 h, respectively, and 3.38- and 3.36-fold higher than that at 24 h, respectively (Fig. 6b). It is noteworthy that the T-MLNs of the 5K-HA-HPPS group had a strong DiR-BOA fluorescence signal for 24 h, and the contralateral N-LNs showed a similar trend but with much lower signal intensity. There was no significant difference in terms of the fluorescence intensity of DiR-BOA between T-MLNs and contralateral N-LNs at 24 h in the HPPS and 5K-HA-e groups (Fig. 6b). This may be due to the uptake of 5K-HA-HPPS by metastatic tumour cells in LNs and the subsequent long period of retention in LNs. Ex vivo fluorescence imaging of 5K-HA-HPPS in resected LNs confirmed the significant difference in fluorescence intensity of DiR-BOA between ipsilateral T-MLNs and contralateral N-LNs (Fig. 6c). The extensive infiltration of tumour cells to the ipsilateral node was also confirmed by the enlarged size of the node (Fig. 6c) and H&E staining of pLNs (Fig. 6d). We also established an orthotopic 4T1 breast tumour model to assess the ability of 5K-HA-HPPS to map metastatic LNs. In addition to primary tumours (yellow arrows), strong fluorescence intensity was seen in the brachial and axillary LNs of mice (yellow circles). Whole-body fluorescence imaging confirmed higher DiR-BOA fluorescence intensities for brachial and axillary LNs in the 5K-HA-HPPS group than in the HPPS group in vivo and ex vivo (Supplementary Fig. S7a, b). These results indicated that dual-targeting by 5K-HA-HPPS permits the mapping of SLNs and discriminates between metastatic and normal LNs through enhanced uptake and retention.

### Inflammation-induced LNs highlighted by 5K-HA-HPPS

**Fig. 7:**
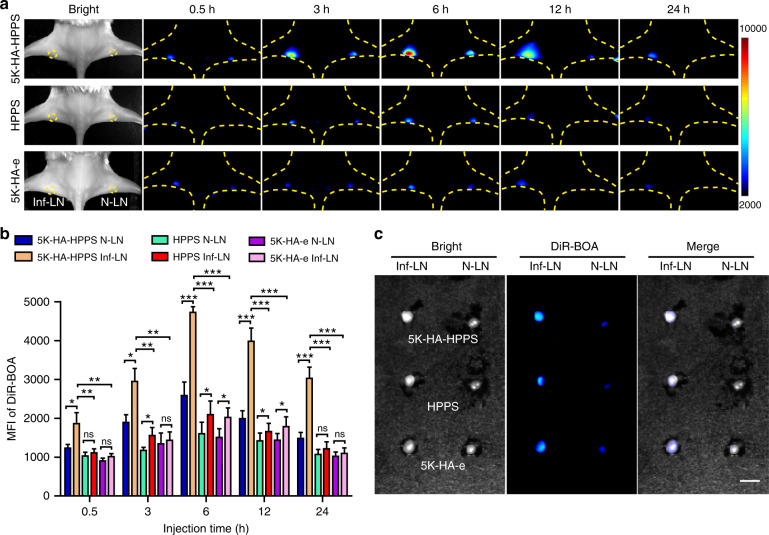
Accumulation of 5K-HA-HPPS in lipopolysaccharide-induced inflamed LNs. **a** Comparison of 5K-HA-HPPS, HPPS and 5K-HA-e signals in the LPS-induced inflammation pLN model (Inf-LN, left) and normal LNs (N-LN, right) by fluorescence imaging at different time points (0.5, 3, 6, 12 and 24 h) after intradermal footpad injection of nanoparticles. **b** Quantitative analysis of the mean fluorescence intensity of DiR-BOA in Inf-LNs and N-LNs. **c** Fluorescence imaging was performed on resected pLNs. Scale bars: 2 mm. Data are presented as the mean ± SD, *n* = 6 mice/group (two-tailed *t*-test)

To determine the ability of 5K-HA-HPPS to distinguish between inflamed LNs (Inf-LNs) and N-LNs, we established an inflammatory LN model by intra-lymph node injection of lipopolysaccharide (LPS) into left pLNs (*n* = 5). As shown in Fig. 7a, due to inflammatory stimulation, left Inf-LNs showed an obviously higher level of tracer accumulation than the contralateral N-LNs in the 5K-HA-HPPS group. Quantification of the fluorescence images showed that the MFI of DiR-BOA in the left Inf-LNs was 2.7- and 2.5-fold higher than that in the contralateral N-LNs at 6 and 24 h, respectively (Fig. 7b) but was weaker than that in T-MLNs (Supplementary Fig. S8a). Moreover, the MFI of DiR-BOA in the 5K-HA-HPPS group was 2.43- and 2.5-fold higher than that in the left Inf-LNs of the HPPS and 5K-HA-e groups at 6 h, respectively. Although left Inf-LNs showed somewhat higher fluorescence intensity than the contralateral N-LNs in the HPPS and 5K-HA-e groups, no significant difference was found, except at 6 and 12 h (Fig. 7a, b). Ex vivo fluorescence images 24 h after injection showed that LPS-induced Inf-LNs were larger than N-LNs and had stronger signals, particularly in the 5K-HA-HPPS group (Fig. 7c). Immunofluorescence imaging of Inf-LNs showed that 5K-HA-HPPS is mainly taken up by macrophages (F4/80^+^) and partially by lymphatic endothelial cells (LECs, Lyve-1^+^) and dendritic cells (DCs, CD11c^+^) but rarely taken up by B cells (B220^+^) and T cells (CD3^+^) (Supplementary Fig. S8b). These results demonstrated that 5K-HA-HPPS is an effective tool for the detection of inflammation-induced LNs through enhanced signal intensity.

### In vivo photoacoustic imaging for predicting the metastatic status of SLNs

**Fig. 8:**
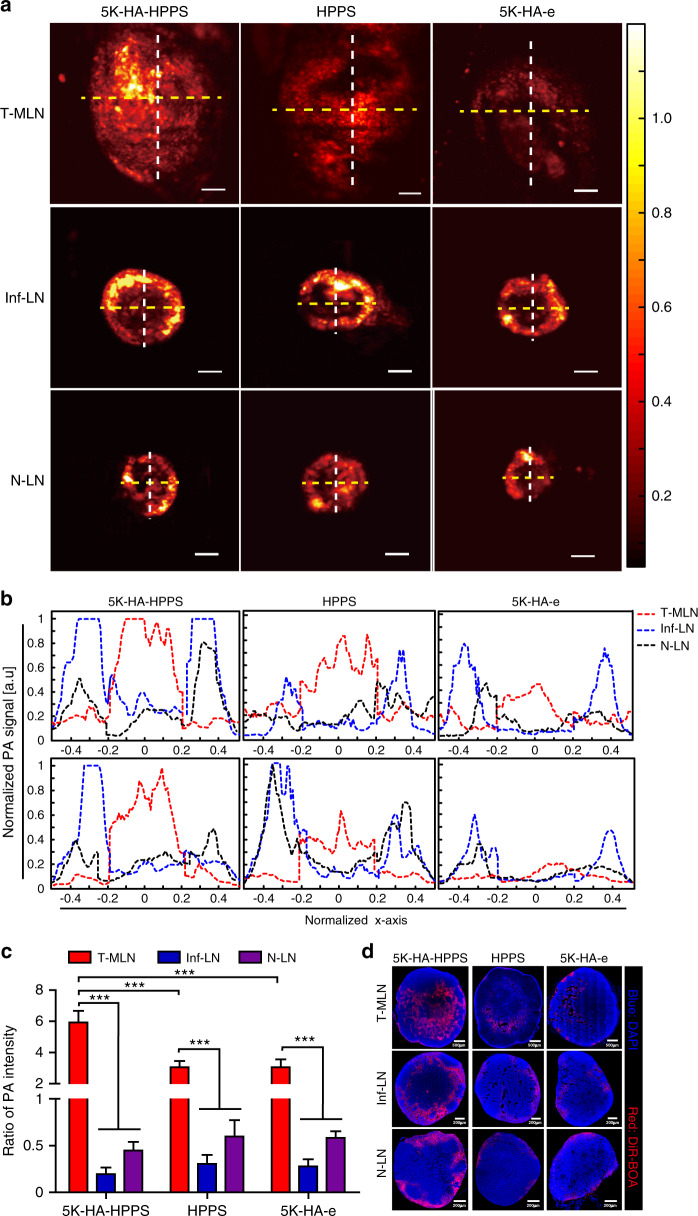
In vivo PAM of 5K-HA-HPPS in LNs with different statuses. **a** In vivo comparison of the distribution of 5K-HA-HPPS (left), HPPS (middle) and 5K-HA-e (right) in 4T1-related SLNs (upper), inflamed LNs (middle) and normal LNs (lower) obtained using the AR-PAM system at 6 h after intratumoural injection (left) or footpad injection (right). Scale bar: 500 μm. *n* = 6 per group. **b** Normalised PA signals of 5K-HA-HPPS, HPPS and 5K-HA-e for distribution and intensity profiles that were taken from the transverse (yellow line) and longitudinal (white line) diameters of each LN depicted in **a**. To quantitatively analyse the distribution of PA signals, the diameters of the LNs with different statuses were normalised, and their centres were designated as the zero position. **c** The ratio of PA intensities (*R* values) at the centre of the LNs to those at their periphery. Data are presented as the mean ± SD, *n* = 6 mice/group (two-tailed *t*-test). **d** After in vivo PAM of LNs with different statuses, frozen sections were taken and confocal microscopy was performed. Blue: DAPI, red: DiR-BOA. Slices: 10 μm

The preceding results demonstrated that both metastatic and inflamed LNs exhibit significantly enhanced nanoparticle uptake, so it is not easy to distinguish between these LNs by fluorescence imaging alone. PAM is a rapidly developing imaging technology for both preclinical and clinical applications. Based on differences in absorbance and the inherent advantages of high-contrast optical imaging and deep ultrasound imaging depth, PAM has been implemented for multiscale imaging from acoustic resolution to optical resolution^29–31^. Given that DiR-BOA can be used for PAM as well as fluorescence imaging, we anticipated that 5K-HA-HPPS(DiR-BOA) can effectively distinguish metastatic SLNs from inflammatory LNs according to the spatial distribution of the PA signal. We performed in vivo PAM for exposed pLNs 6 h after hock injection of nanoparticles. As shown in Fig. 8a, there were strong PA signals at the peripheries of N-LNs and Inf-LNs in the 5K-HA-HPPS group, whereas the signals within the LNs were very weak. Interestingly, the opposite trend was seen in T-MLNs, which had strong PA signals within the SLNs (Fig. 8a, and Movies S1–S3). Although the PA signals of the LNs in the HPPS and 5K-HA-e groups exhibited the same trend in terms of distribution as T-MLNs in 5K-HA-HPPS, their signal intensities were much weaker (Fig. 8a). To quantitatively analyse the distribution characteristics of the PA signals in LNs with different statuses, we extracted PA signals along the transverse (yellow line) and longitudinal (white line) diameters of each LN and drew cross-sectional intensity profiles (Fig. 8b). The diameters of the LNs with differing statuses were normalised, and the centre of each LN was denoted as the zero position (Supplementary Fig. S9). The ratios (*R* values) of PA intensities at the centre of the LNs to those at their peripheries were 5.93 ± 0.75 for T-MLNs of the 5K-HA-HPPS group, which was much higher than that for Inf-LNs (*R* = 0.2 ± 0.07) and N-LNs (*R* = 0.45 ± 0.09). Although the intensity profiles of the HPPS and 5K-HA-e groups were similar to that of the 5K-HA-HPPS group, the ratios of PA intensity for T-MLNs of the HPPS (*R* = 3.06 ± 0.4) and 5K-HA-e (*R* = 3.05 ± 0.5) groups were ~50% of that of the 5K-HA-HPPS group (*R* = 5.93 ± 0.75) (Fig. 8c). The characteristic distribution patterns of nanoparticles in the LNs with different statuses were also confirmed by confocal microscopy (Fig. 8d). Moreover, we performed PAM on earlier T-MLNs at 1 and 2 weeks after tumour inoculation, and distribution of the PA signals was found to concur with those SLNs at 3 weeks of metastasis (Supplementary Fig. S10). Thus, 5K-HA-HPPS combined with PAM can not only distinguish T-MLNs from Inf-LNs and N-LNs but can also be applied to detect early T-MLNs, guiding clinicians in removing SLNs during breast cancer surgery.

## Discussion

LNs are among the most important organs of the immune system and play critical roles in not only removing pathogens but also producing lymphocytes and antibodies. SLNs are LNs in which a primary tumour first metastasises. Accurate detection and characterisation of SLNs are crucial for cancer staging and making therapeutic decisions, particularly in patients with breast cancer or melanomas^32,33^. At present, the clinical gold standard used to detect SLNs is labelling with blue dye or a radioactive nanocolloid and then performing SLNB. Although various nanoprobes based on different imaging modalities have been developed to map the metastatic status of SLNs^12–15,34–36^, few can distinguish T-MLNs from Inf-LNs. Tumour cell infiltration and proliferation of immune cells due to the induction of inflammatory factors result in the enlargement of SLNs^37^, usually leading to misidentification and unnecessary removal of benign SLNs during surgical resection^38^. Therefore, whether SLN enlargement is caused by the metastasis of tumour cells or inflammation should be determined before SLN resection to reduce potential complications caused by unnecessary resection.

Breast cancer cells are well known to overexpress CD44 proteins. Recently, Tao et al. reported that SR-B1 is also expressed on breast cancer cells^26^. Previously, we developed an HPPS nanoparticle with strong SR-B1 targeting ability^24^. By incorporating HA into HPPS, we established a novel dual-targeting 5K-HA-HPPS that can simultaneously target CD44 and SR-B1. It is well understood that lymphatic endothelial cells in LNs express lymphatic vessel endothelial hyaluronan receptor 1 (Lyve-1), which is a homologue of the CD44 glycoprotein and a lymph-specific receptor that specifically binds HA^39,40^. Yang et al. used HA of different molecular weights (5K/10K/15K) as SLN mapping agents and reported that 10K HA had favourable migration and retention profiles because it binds to Lyve-1 in LNs. The combination of HA and tumour-targeting antibodies (EGFR and HER2) can detect UM-SCC-22B tumour metastasis in SLNs^41^. However, this method of identifying T-MLNs is not ideal because it can only identify partial and fully occupied SLNs due to the minimal blood supply able to deliver the imaging probes to micrometastases and the simultaneous injection of antibody and HA, which should be separated by 24 h. In our study, we found that 5K-HA-HPPS rapidly (<10 min) migrated to pLNs and showed prolonged retention (>12 h) compared with 15K-HA-HPPS. Indeed, fluorescence imaging of LN sections demonstrated that 5K-HA-HPPS can partially bind Lyve-1 within LNs (Supplementary Fig. S11). In addition to 4T1 cells, we demonstrated that 5K-HA-HPPS has the same targeting effect in B16F10 melanoma cells and CT26 colorectal cancer cells (Supplementary Fig. S7), suggesting that dual-targeted 5K-HA-HPPS can be applied to a variety of tumour cells. Due to the excellent scalability of the nanocarrier HPPS, it can be loaded with different imaging contrast agents to detect the migration of immune cells^42^ and can also carry drugs (e.g. curcumin^43^) for experimental autoimmune encephalomyelitis treatment or carry immune adjuvants (e.g. CpG^21^) for tumour treatment. In this study, the nanoparticles were loaded with molecular HA. We can also change the targeting ligand according to different biomarkers (e.g. EGFR, folate receptor) expressed by tumours^24^. Therefore, this novel nanoparticle probe may provide better diagnostic imaging and therapeutic potential for the detection and treatment of serious diseases.

LN enlargement can occur during both tumour cell invasion and under inflammatory conditions. Recently, various imaging agents have been developed to identify the metastatic status of SLNs based on indirect mapping agents such as single-walled carbon nanotubes^44^, albumin^45^ and upconversion nanoparticles^46^. The use of these indirect mapping agents is not the best means to distinguish T-MLNs because it is difficult to detect SLNs that are completely occupied by tumour cells. Hence, the ideal mapping agent can directly target tumour cells and distinguish T-MLNs from Inf-LNs. In our study, the fluorescence signal of 5K-HA-HPPS in enlarged Inf-LNs was enhanced compared with that in N-LNs. This may be attributed to the proliferation of macrophages and lymphangiogenesis caused by inflammatory stimulation, thus resulting in enhanced accumulation of 5K-HA-HPPS in Inf-LNs. Fluorescence imaging showed that 5K-HA-HPPS accumulated in both T-MLNs and Inf-LNs (Figs. 6, 7), indicating that T-MLNs and Inf-LNs cannot be distinguished according to their fluorescence intensities. However, we found that 5K-HA-HPPS can effectively target 4T1 cells in metastatic LNs.

PAM is a rapidly developing imaging technology, and all types of nanoscale agents have been comprehensively explored as PA imaging probes to map SLNs^34,47–49^. Luke et al. introduced EGFR-targeted plasmonic nanosensors (MAPS), which can noninvasively and effectively identify oral squamous cell carcinoma sLN micrometastases from normal LNs by using ultrasound-guided spectroscopic photoacoustic (sPA) imaging^50^. This method has great potential for clinical translation due to combined ultrasound imaging, sPA imaging and MAPS. Previously, we developed a self-assembled nano-pomegranate with the NIR dye DiR-BOA, which has fluorescence/photoacoustic imaging-switching (‘off/on’) capability^28^. Therefore, we attempted to distinguish the different statuses of LNs using PAM. Remarkably, we found that PA signals from 5K-HA-HPPS showed a significantly distinct spatial distribution among LNs of different statuses. This result suggests that 5K-HA-HPPS possesses a potent ability to distinguish T-MLNs from Inf-LNs and N-LNs. The potential reason for the spatial differences of 5K-HA-HPPS in T-MLNs and Inf-LNs is the difference in targeting efficiencies between tumour cells and macrophages. Our results showed that 5K-HA-HPPS is mainly taken up by macrophages in inf-LNs and partially by LECs and DCs (Supplementary Fig. S8b), while it more effectively targets metastatic 4T1 cells in the inner part of T-MLNs than cells in the peripheral region of T-MLNs in vivo (Fig. 5d). The PA signal of 5K-HA-HPPS was weak in early T-MLNs (1–2 weeks) (Supplementary Fig. S10), perhaps because fewer tumour cells metastasised into SLNs, but the distribution was still different from that in Inf-LNs and N-LNs.

In summary, we developed a novel dual-targeting agent, 5K-HA-HPPS, with fluorescence/photoacoustic dual-modal imaging capability. Wide-field fluorescence imaging showed that 5K-HA-HPPS can effectively map pLNs and that the fluorescence intensities of the nanoparticles were enhanced in T-MLNs and Inf-LNs. Surprisingly, PA showed a reverse spatial distribution in LNs with different statuses, whereby the PA signals of 5K-HA-HPPS were mainly distributed within T-MLNs but at the peripheries of Inf-LNs and N-LNs because 5K-HA-HPPS can effectively target metastatic tumour cells in T-MLNs. Thus, 5K-HA-HPPS can provide important guidance for the resection of SLNs in breast cancer surgery and facilitate the implementation of appropriate tumour treatment strategies.

## Materials and methods

1,2-Dimyristoyl-sn-glycero-3-phosphocholine (DMPC), 1,2-dimyristoyl-sn-glycero-3-phosphoethanolamine (DMPE) and 1,2-distearoyl-sn-glycero-3-phosphoethanolamine-N-[methoxy(polyethylene glycol)-2000] (ammonium salt) (DSPE-PEG2000, ammonium salt) were obtained from Avanti Polar Lipids Inc. (Alabaster, AL, USA). HA with different molecular weights of 5 and 15 kDa were obtained from Lifecore Biomedical LLC (MN, USA) (cat#: HA5K-1, HA15K-1). 1-Ethyl-3-[3-dimethyl)aminopropyl]carbodiimide (EDAC) was purchased from Sigma-Aldrich Co. (St. Louis, MO, USA). The near-infra-red dye DiR-BOA (1,1′-dioctadecyl-3,3,3′,3′-tetra-methylindotricarbocyanine iodide bis-oleate) was synthesised as previously described^51^. R4F (an ApoA1-mimetic peptide, Ac-FAEKFKEAVKDYFAKFWD) was synthesised by Shanghai Apeptide Co., Ltd. (Shanghai, China).

### Mice and cells

Albino-C57BL/6 transgenic mice were obtained from the Jackson Laboratory (Bar Harbor, ME). BALB/c female mice were purchased from the Hubei Research Center of Laboratory Animals (Hubei, China). All animal studies were conducted in compliance with protocols that had been approved by the Hubei Provincial Animal Care and Use Committee and in compliance with the experimental guidelines of the Animal Experimentation Ethics Committee of Huazhong University of Science and Technology (IACUC number: 2160). The mouse mammary adenocarcinoma 4T1 cell line was kindly provided by Professor Li Su (Huazhong University of Science and Technology). 4T1-tfRFP cells were obtained by transfecting 4T1 cells with a plasmid containing the tfRFP gene^52^. The B16F10 cell line was purchased from BOSTER Company (Wuhan, China), and the CT26 cell line was obtained from the American Type Culture Collection (Manassas, USA). These cells were cultured in complete RPMI-1640 medium (Gibco, Thermo Fisher Scientific, USA) containing 10% foetal bovine serum (FBS, Gibco) and 1% penicillin–streptomycin (Gibco) in a cell incubator (Thermo, USA) with 5% CO_2_ and 95% air at 37 °C.

### Synthesis of HA-DMPE

HA-DMPE was synthesised as described previously using a modified reaction^53^. Briefly, 14 mg of HA was dissolved in 5 ml of distilled water, and 6 mg of EDAC was added at pH 4 to preactivate the HA (5 and 15 kDa) for 2 h at 37 °C. Subsequently, a suspension of DMPE (0.5 mg) was added to the preactivated HA solution, and the pH was adjusted to 8.6 with 0.1 M borate buffer (pH 9.4). This reaction proceeded for 24 h at 37 °C. The mixture was purified by dialysis (MWCO 3 kDa) four times for 12 h each time. Then, the purified solution was lyophilised, and 1 ml of chloroform–methanol (4:1; v/v) was added to the lyophilised sample. Next, the sample was centrifuged at 12,000 rpm for 10 min at 4 °C; this step was repeated three times. The free HA precipitate was removed, and the supernatant of the HA-DMPE solution was collected and stored at 4 °C. The structures of HA, DMPE and the HA-DMPE copolymers were determined using ^1^H NMR (AV400, Bruker, Switzerland) in D_2_O and DMSO-*d*_6_.

### Synthesis and characterization of the HA-HPPS core loaded with DiR-BOA

HA-HPPS complexes were synthesised as follows. DMPC (3 μmol), cholesterol oleate (CO, 0.1 μmol), DiR-BOA (0.2 μmol), DSPE-PEG2000 (0.0114 μmol) and HA-DMPE (5 or 15 kDa, 0.04 μmol) in chloroform (400 μl) were dried under a nitrogen stream to form a uniform film. Then, 2 ml of PBS solution was added to the dried film and vortexed for 5 min. Subsequently, the mixture was sonicated for ~1 h at 48 °C. R4F (0.78 μmol) was dissolved in 1 ml of PBS, added dropwise to the lipid emulsion and stored overnight at 4 °C. After concentration using centrifugal filter units (30 kDa, Millipore, USA), the nanoparticles were purified using the Akta FPLC system with a HiLoad 16/70 Superose 6 column (General Electric Healthcare, NY, USA). The peptide concentration was measured using a CBQCA protein quantitation kit (Invitrogen Corporation, CA, USA). The morphologies of the nanoparticles were analysed by TEM (TECNAI G2, FEI Company, OR, USA). The size distributions of the nanoparticles were measured by DLS on a Zetasizer Nano-ZS90 (Malvern Instruments, Worcestershire, UK). The stability of the nanoparticles was evaluated using seminative SDS–PAGE.

### Cytotoxicity test

The cytotoxicity of the samples to 4T1 cells was determined by the MTS assay. In brief, 1 × 10^4^ 4T1 and RAW264.7 cells (in 100 μl of medium) were seeded in each well of a 96-well culture plate and incubated at 37 °C with 5% CO_2_ for 24 h. The medium in each well was replaced with culture medium (100 μl) containing different concentrations (1–40 μM) of HA-HPPS. After incubation for 24 h, 20 μl of MTS was added to each well, and incubation was continued for 1 h at 37 °C. Then, absorption at 490 nm was measured using a Bio-Tek Epoch microplate spectrophotometer (Winooski, Vermont, USA).

### Confocal imaging

To illustrate the 4T1 cell dual-targeting ability of the nanoparticles in vitro, 4T1 cells, RAW264.7 cells, BMDMs and BMDCs were seeded into 8-well chambers covering the glass bottoms (Nunc Lab-Tek, Thermo Scientific) (2 × 10^4^ cells/well). Then, the cells were incubated with 5K-HA-HPPS, HPPS or 5K-HA-e at a DiR-BOA concentration of 10 μM for 3 h, and Hoechst 33258 (0.5 μg ml^−1^) was added 15 min before washing. For imaging of the tissue sections, the LNs were fixed in 4% paraformaldehyde for 10 h at 4 °C and then dehydrated in a 30% sucrose solution. The LNs were then frozen in OCT compound (Sakura, Torrance, CA, USA) and sectioned into 10-μm-thick slices using a freezing microtome (Leica, Germany). For staining, slides were washed once with PBS, and the sections were immunostained with Lyve-1 (eBioscience, clone ALY7, 1:200), F4/80 (Clone: BM8, 1:200), CD11c (Clone: N418, 1:100), CD3 (Clone: 17A2, 1:200) and B220 (Clone: RA3-6B2, 1:200). Fluorescence images were acquired using an LSM 710 laser confocal scanning microscope (Zeiss, Germany) with an excitation wavelength of 405 nm for Hoechst 33258 and DAPI, 488 nm for FITC and Lyve-1 and 633 nm for DiR-BOA.

### FCM analysis

For 4T1 cell-targeting ability testing, 4T1 cells, B16 cells, CT26 cells and E0771 cells were plated in 96-well flat-bottom culture plates (5 × 10^4^ cells/well), and 5K-HA-HPPS, HPPS or 5K-HA-e were incubated with the cells at various DiR-BOA concentrations for 0.5 or 3 h. Regarding the competition assay, 4T1 cells were precultured with excessive amounts of HDL protein and free HA. The mass ratio of HDL to R4F in 5K-HA-HPPS and HPPS was 10:1 and that of free HA to HA in 5K-HA-HPPS and 5K-HA-e was 250:1. Then, the cells were incubated with 5K-HA-HPPS, HPPS or 5K-HA-e at a DiR-BOA concentration of 10 μM for 3 h. The total volume of the solution in the wells was 200 μl for FCM. Fluorescence signals were quantified by a Guava easyCyte 8HT flow cytometer (Millipore Corporation, Billerica, MA, USA). The data were analysed using FlowJo.

### LN metastasis and inflammation models

To establish the LN metastasis model of 4T1 murine breast cancer, 5 × 10^5^ 4T1 cells or 4T1-tfRFP cells in 20 μl of PBS were injected into the left hock area of BALB/c mice. Albino-C57BL/6 mice were used to develop the pLN inflammation model by intra-lymph node injection of LPS (5 ml kg^−1^, Sigma) 2 days before imaging experiments^11^. In vivo imaging experiments were performed using a custom-made whole-body optical imaging system.

### In vivo fluorescence imaging

For in vivo fluorescence imaging of the LNs, 25 nmol of 5K-HA-HPPS, HPPS or 5K/15K-HA-e were injected into one side of the footpad of normal Albino-C57BL/6 mice, and nanoparticle accumulation in pLNs and sLNs was detected and analysed by wide-field fluorescence imaging at 10 min, 1, 3, 6 and 12 h after injection of the probe. For fluorescence imaging of Inf-LNs and T-MLNs, 5K-HA-HPPS, HPPS or 5K/15K-HA-e were injected into both sides of the footpad of inflammatory Albino-C57BL/6 mice and the hock area of BALB/c mice. The fluorescence signals of N-LNs, T-MLNs and Inf-LNs were imaged at 0.5, 3, 6, 12 and 24 h after injection of the probes. Fluorescence images of DiR-BOA were acquired with an NIR filter set (excitation: 716/40 nm; emission: 800/40 nm; exposure time: 10 or 30 s). The mice were anaesthetised with 3% isoflurane/O_2_ (v/v) and maintained on isoflurane/O_2_ at 1.5% (v/v) throughout the experiments. For LN imaging, 3 weeks after hock inoculation of 4T1-tfRFP tumour cells, resected T-MLNs were imaged 6 h after intratumoural injection of FITC–5K-HA-HPPS and FITC–HPPS. Fluorescence images of tfRFP and FITC were acquired with a filter set (excitation: 562/40 nm, emission: 640/40 nm; and excitation: 496/40 nm, emission: 562/40 nm, respectively). Regarding the orthotropic breast cancer model, tumour-bearing mice were subjected to fluorescence imaging on day 30 after tumour inoculation (*n* = 3). A single dose of 5K-HA-HPPS or HPPS (DiR-BOA: 25 nmol) in 50 μl of sterile PBS was injected intratumourally. Imaging of brachial and axillary LNs was performed 6 h after injection of the probes.

### In vitro and in vivo PAM

For in vitro PAM of 4T1 cells and BMDMs, 4T1 cells and BMDMs were seeded into 15 mm dishes and incubated with 5K-HA-HPPS, HPPS or 5K-HA-e for 3 h in vitro.

For in vivo PAM analysis of different statuses of LNs, the skin was excised and pLNs were exposed 6 h after injection of 5K-HA-HPPS, HPPS or 5K-HA-e (DiR-BOA: 25 nmol). Photoacoustic (PA) maximum amplitude projection images (image field of view: 5 mm × 5 mm) were taken in vivo with the custom-made acoustic resolution PAM (AR-PAM) system with 744 nm pulsed lasers at 600 nJ. The images were acquired by performing a B-scan, with each B-scan including 500 steps with a step size of 10 μm. The maximum image depth of AR-PAM was 1.6 mm. The spatial resolution of AR-PAM was ~45 μm. To quantitatively analyse the distribution of PA signals in LNs with different statuses, we extracted PA signals along the transverse (dotted yellow line) and longitudinal (dotted white line) diameters of each LN and constructed cross-sectional intensity profiles to reduce error from single section measurement. The LN centre (C) was defined as 40% of the diameter (20% on each side of the centre point). The LN periphery (P) was defined as 60% of the diameter (30% on each side of the edge). The average ratio of PA intensity in the LN centre (C) to that of the LN periphery (P) was calculated by the formula *R* = (*R*_T_ + *R*_L_)/2. *R*_T_ and *R*_L_ represent the ratio of transverse (T) and longitudinal (L) PA intensity, respectively, and were calculated as follows:

$$R_T = C_1/\left( {\frac{{P_1 + P_2}}{2}} \right),$$

$$R_L = C_2/\left( {\frac{{P_3 + P_4}}{2}} \right),$$

where *C*_1_ and *C*_2_ represent the PA intensities of the LN centre in the transverse and longitudinal sections, respectively; *P*_1_ and *P*_2_ represent the PA intensities of the LN periphery in the transverse section on the left and right sides, respectively; and *P*_3_ and *P*_4_ represent the PA intensities of the LN periphery in the longitudinal section on the top and bottom, respectively (Supplementary Fig. S9).

### Statistical analysis

Statistical analysis was performed using GraphPad Prism 5 (GraphPad Software, CA). Student’s *t* test (two tailed) was used for the in vitro and in vivo studies. Data are presented as the mean ± SD. Significant differences between or among the groups are indicated as follows: ns for no significant difference, * for *P* < 0.05, ** for *P* < 0.01, and *** for *P* < 0.001.

## Supplementary information

Supplementary material

5K-HA-HPPS in lnf-LN

5K-HA-HPPS in N-LN

5K-HA-HPPS in T-MLN
