# Supplementary material for: Metastatic status of sentinel lymph nodes in breast cancer determined with photoacoustic microscopy via dual-targeting nanoparticles
Source: Light Sci Appl. 2020 Sep 16;9:164. doi: 10.1038/s41377-020-00399-0 (PMC7494891; doi:10.1038/s41377-020-00399-0)
Supplement: Supplementary file 1 — Supplementary material [file 41377_2020_399_MOESM1_ESM.docx]

Supplementary Information for

**Metastatic Status of Sentinel Lymph Nodes in Breast Cancer Determined with Photoacoustic microcopy via Dual-Targeting Nanoparticles**

Yanfeng Dai^1,2,#^, Xiang Yu^1,2,#^, Jianshuang Wei^1,2,#^, Fanxin Zeng^1,2^, Yiran Li^2^,

Xiaoquan Yang^1,2^, Qingming Luo^1,2,3,*^, Zhihong Zhang^1,2,3,*^

This PDF file includes:

- Fig. S1. Synthesis of HA-DMPE conjugate.
- Fig. S2. *In vitro* characterization of HA-HPPS.
- Fig. S3. Wide-field fluorescence imaging for the capability of 5K-HA-HPPS migrate to pLNs.
- Fig. S4. *In vivo* comparison of the migration of 15K-HA-HPPS, HPPS, and 15K-HA-e to pLNs.
- Fig. S5. Flow cytometry the uptake of 5K-HA-HPPS by B16 cells, CT26 cells and E0771 cells.
- Fig. S6. 5K-HA-HPPS efficiently targeted to 4T1 cells *in vitro*.
- Fig. S7. Whole-body fluorescence imaging of orthotropic tumor-draining LNs.
- Fig. S8. *In vivo* comparison of fluorescence intensity of 5K-HA-HPPS in T-MLNs and Inf-LNs and confocal imaging 5K-HA-HPPS uptake by immune cells in Inf-LN.
- Fig. S9. Schematic for quantification the PA signal for LNs with different statuses.
- Fig. S10. PAM of SLNs in early tumour metastasis.
- Fig. S11. Confocal microscopy of 5K-HA-HPPS binding with Lyve-1.


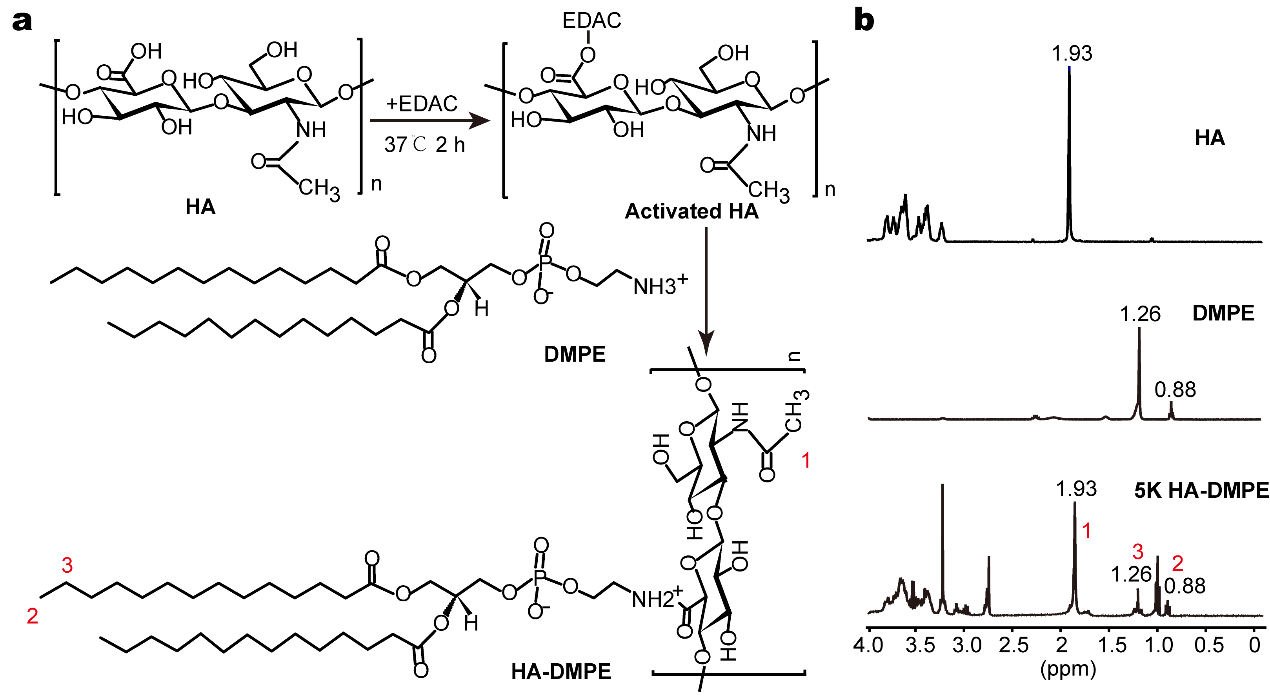


**Fig. S1.** **Synthesis of HA-DMPE conjugate** (**a**) Schematic of the activation of HA carboxylic acid groups with EDAC and the conjugation of DMPE to the HA backbone. (**b**) The ^1^H NMR spectrum of HA in D_2_O, DMPE and 5K-HA-DMPE in DMSO-*d*_6_.

**
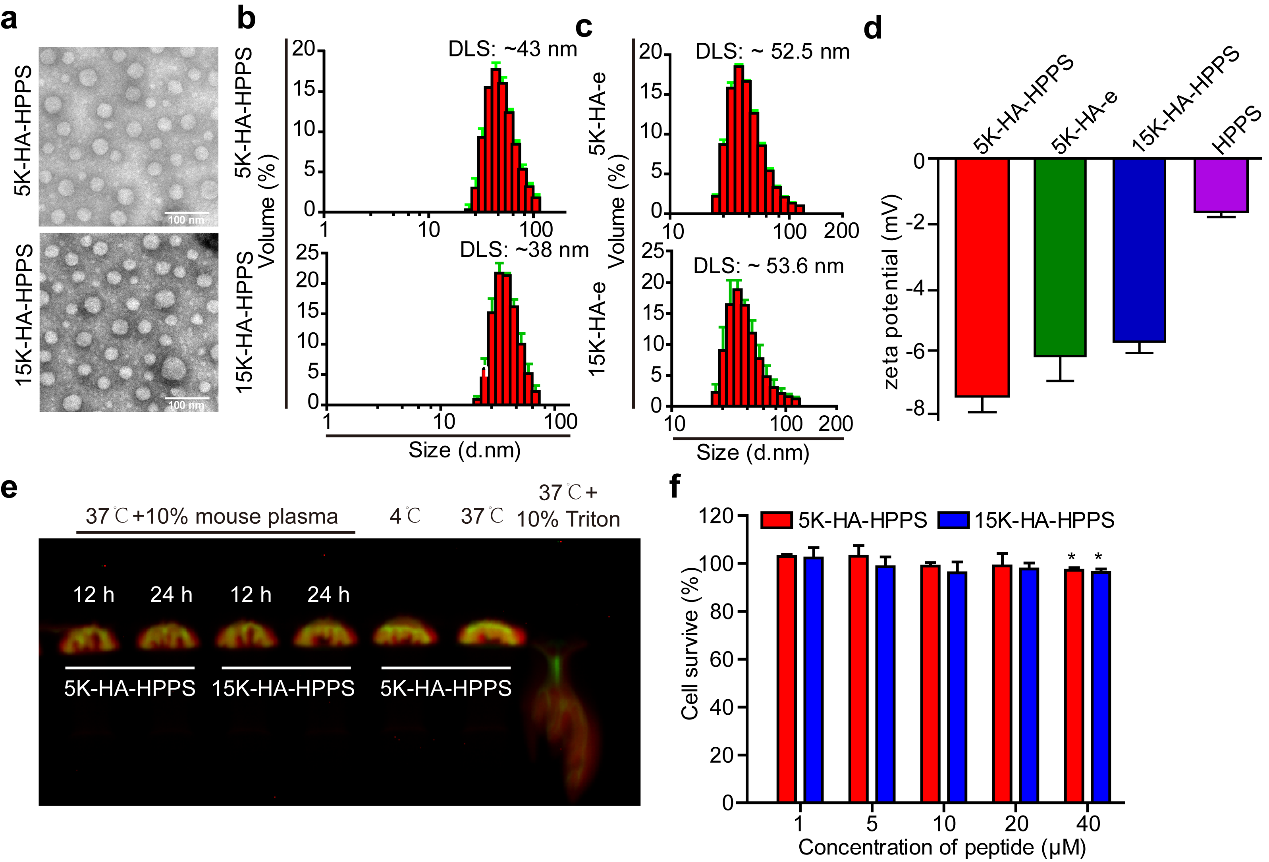
**

**Fig. S2.** ***In vitro* characterization of HA-HPPS.** (**a**) TEM images of HA-HPPS. (**b**) Size distribution of HA-HPPS detected using dynamic light scattering (DLS). (**c**) Size distribution of HA emulsions (5K-HA-e and 15K-HA-e) measured by DLS. (**d**) Zeta potential measurements of 5K-HA-HPPS, 5K-HA-e, 15K-HA-HPPS, and HPPS. (**e**) SDS-PAGE and fluorescence imaging to evaluate the stability of FITC–5K-HA-HPPS(DiR-BOA) and FITC–15K-HA-HPPS(DiR-BOA) after for 12 h and 24 h incubation. Red: DiR-BOA, Green: FITC. (**f**) RAW264.7 cells were used to evaluate the cytotoxicity of 5K-HA-HPPS and 15K-HA-HPPS at various concentrations after 24 h incubation. Data are presented as the mean ± SD. *P < 0.05. n = 3.


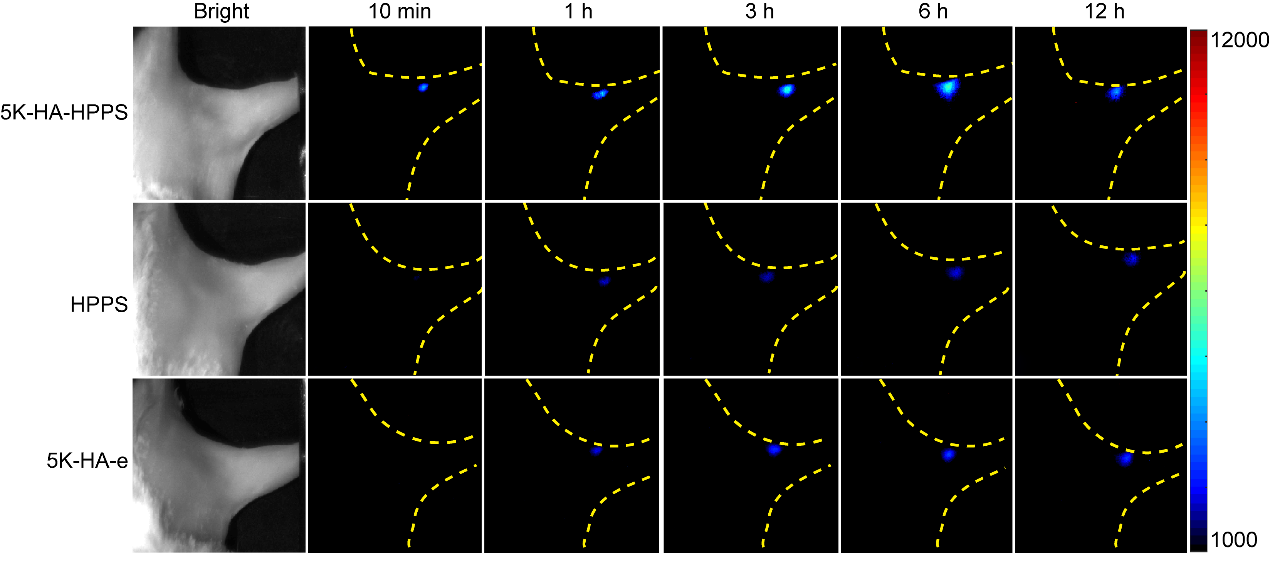


**Fig. S3.** **Wide-field fluorescence imaging for the capability of 5K-HA-HPPS to migrate to pLNs.** After the intradermal injection of 5K-HA-HPPS, HPPS, and 5K-HA-e into the hind footpads of normal albino C57BL/6 mice (n = 5 mice/group), wide-field fluorescence imaging was performed to observe the migration capability at 10 min, 1 h, 3 h, 6 h, and 12 h. Exposure time: 10 s.

**
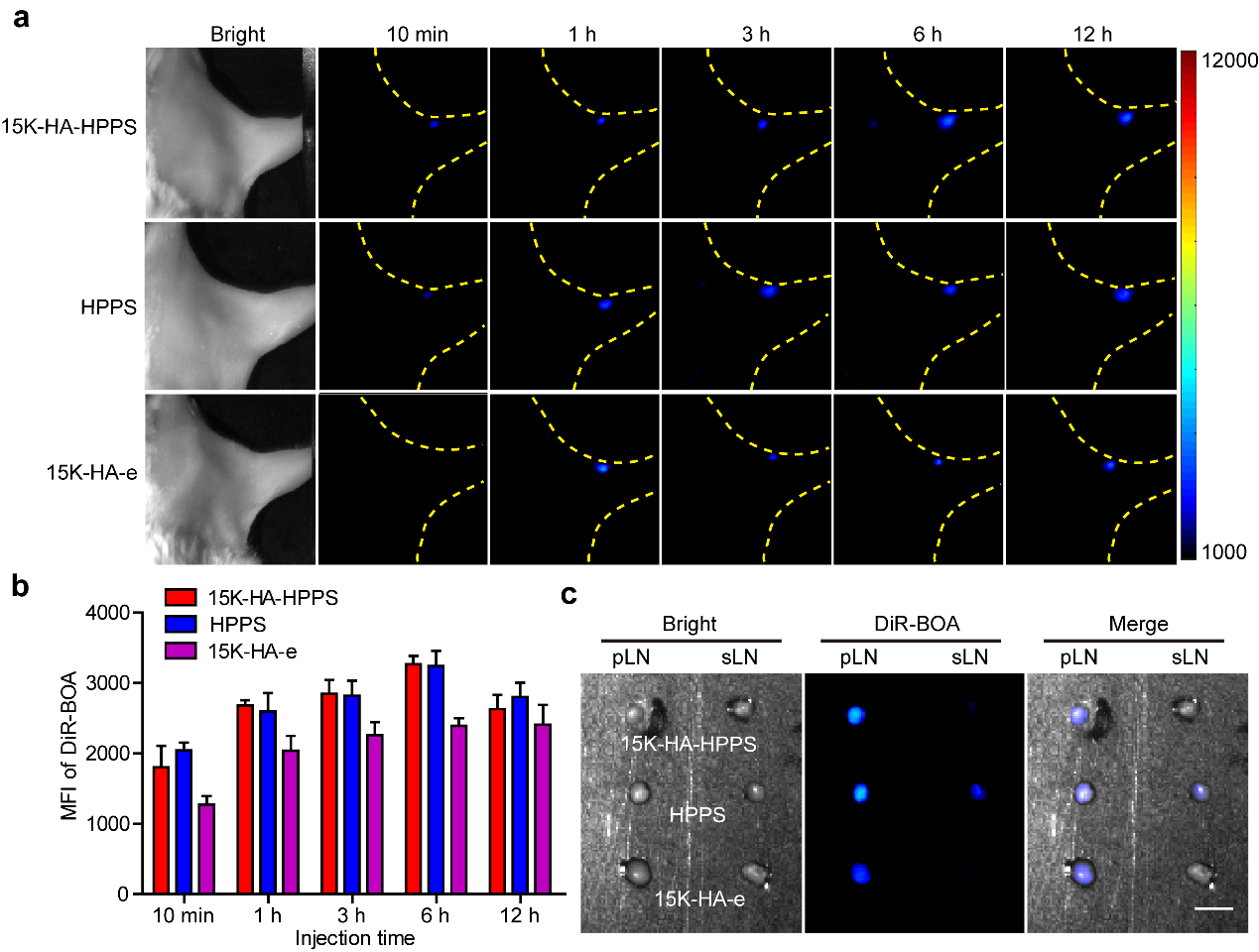
**

**Fig. S4.** ***In vivo* comparison of the migration of 15K-HA-HPPS, HPPS and 15K-HA-e to pLNs.** (**a**) Representative fluorescence imaging was conducted to observe the effect of 15K-HA-HPPS, HPPS, and 15K-HA-e migration to pLNs at 10 min, 1 h, 3 h, 6 h, and 12 h after footpad intradermal injection. Exposure time: 30 s (**b**) Quantitative analysis of the fluorescence intensity of pLNs in (**a**). (**c**) Fluorescence imaging was performed 12 h after injection to resected pLNs and sLNs. Scale bar: 2 mm. Data are presented as the mean ± SD (two-tailed t-test; n = 5 mice/group).

**
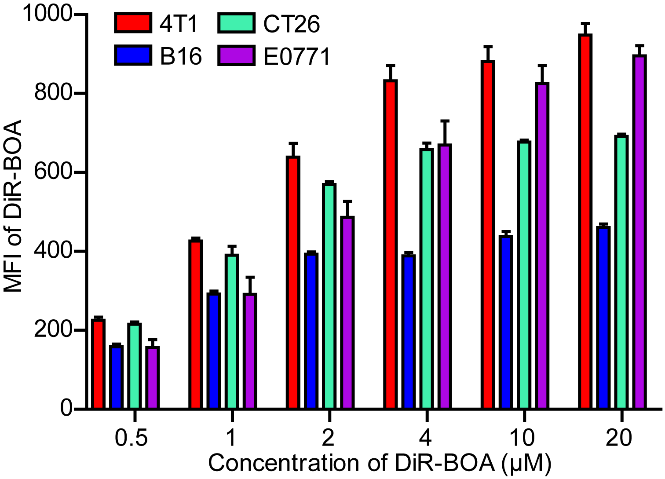
**

**Fig. S5.** **Flow cytometry the uptake of 5K-HA-HPPS by B16 cells, CT26 cells and E0771 cells.** After 3 h incubation, flow cytometry of B16F10 and CT26 cells and E0771 cells uptake of 5K-HA-HPPS at different DiR-BOA concentrations (0.5, 1, 2, 4, 10, and 20 μM). The data are presented as the mean ± SD (n = 3).


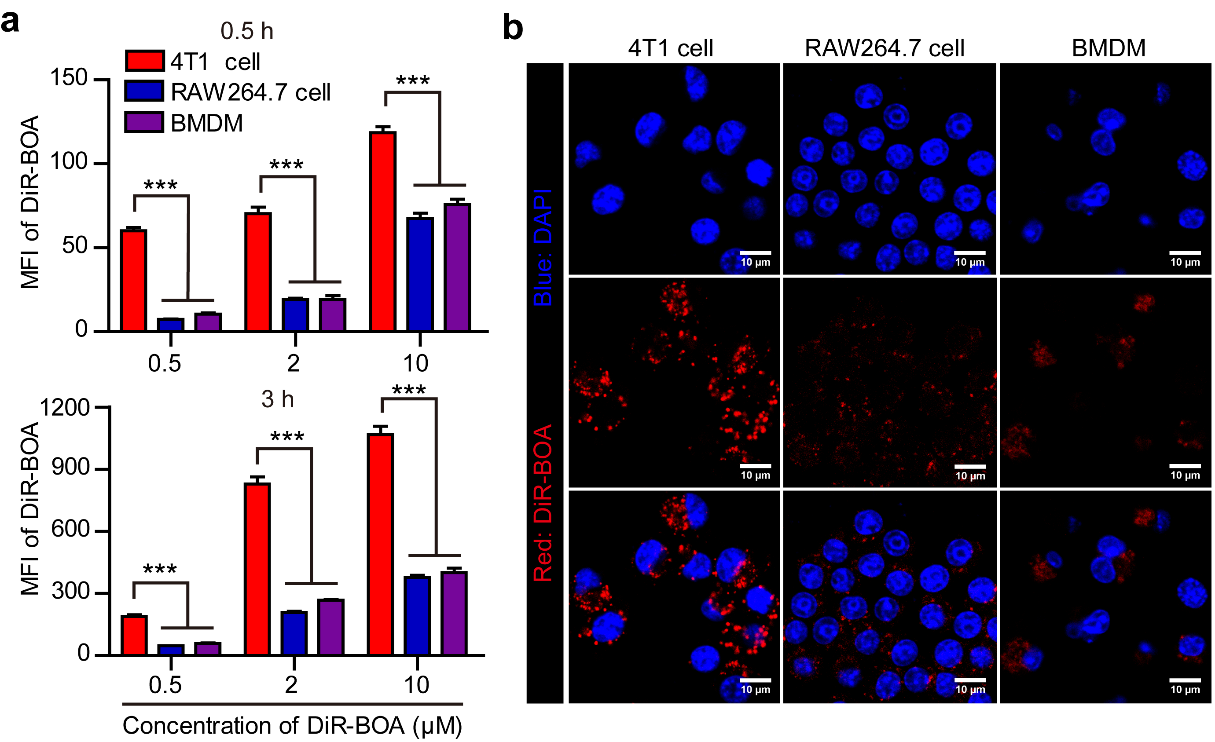


**Fig. S6. 5K-HA-HPPS is efficiently targeted to 4T1 cells in vitro.** (**a**) Flow cytometry was performed to analyze and compare uptake of 5K-HA-HPPS by 4T1 cells, RAW264.7 cells, and BMDM in vitro after exposure to various concentrations for 0.5 h and 3 h. (**b**) Confocal imaging was performed to compare 4T1，RAW264.7, BMDM, and BMDC cell uptake of 5K-HA-HPPS in vitro after exposure to 10 μM DiR-BOA for 3 h incubation. Data are presented as the mean ± SD (two-tailed t-test; n = 3).


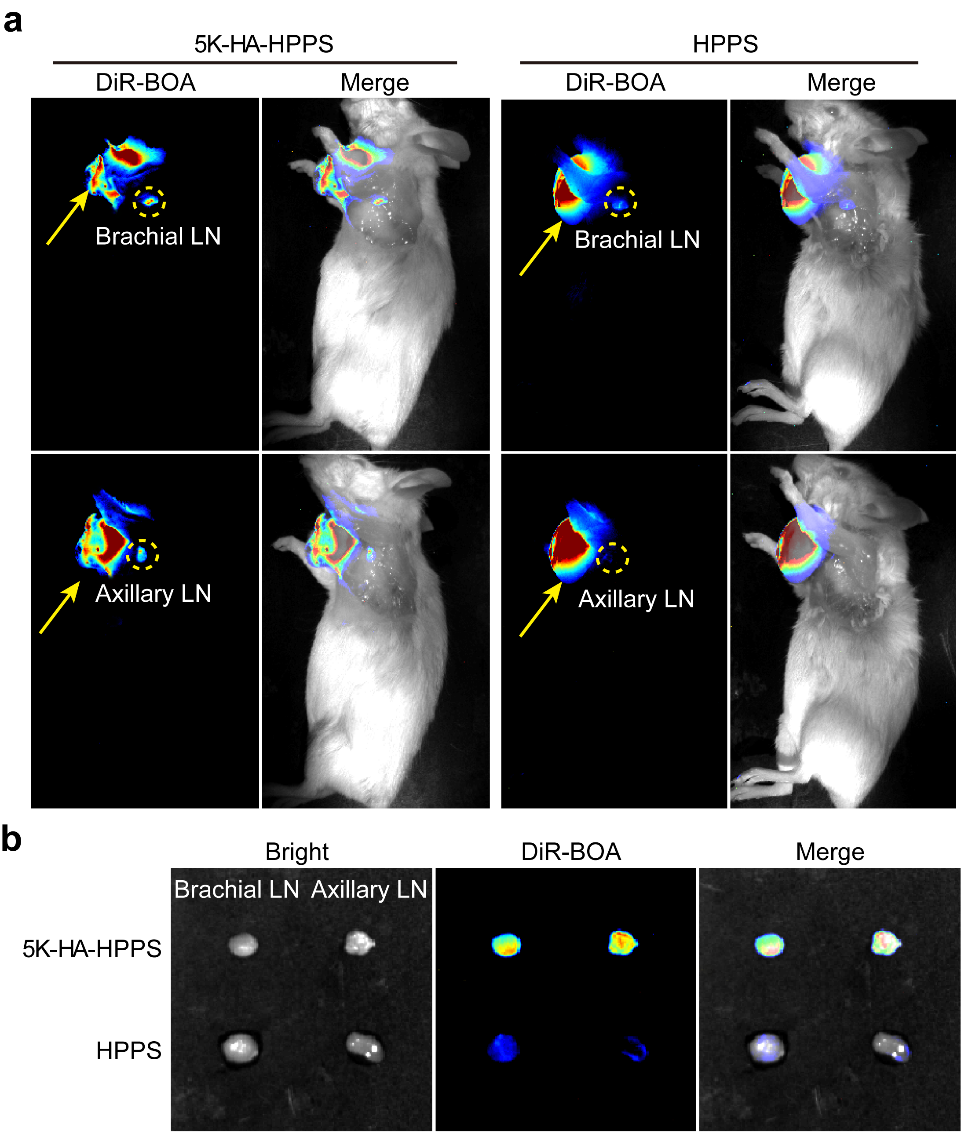


**Fig. S7.** **Whole-body fluorescence imaging of orthotropic tumor-draining LNs.** (**a**) Representative 5K-HA-HPPS and HPPS related whole-body fluorescence images of brachial and axillary LNs (yellow doted circle) in the orthotropic breast cancer model. Images were taken at 6 h after tracer injection; arrowheads indicate primary tumors. n = 3 mice/group. (**b**) *Ex vivo* imaging of resected brachial and axillary LNs.


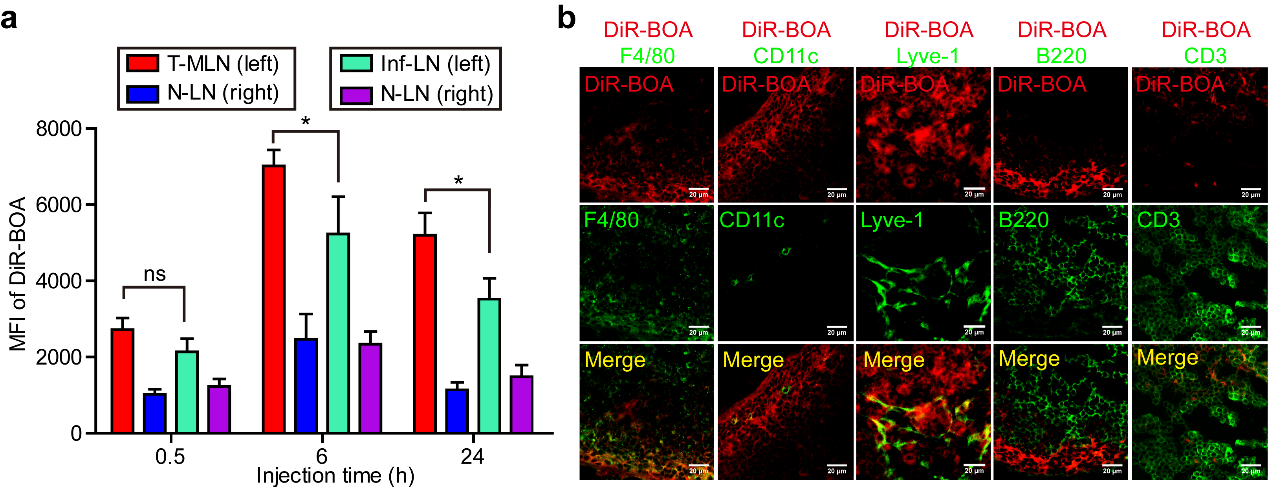


**Fig. S8**. ***In vivo* comparison of fluorescence intensity of 5K-HA-HPPS in T-MLNs and Inf-LNs and confocal imaging 5K-HA-HPPS uptake by immune cell in Inf-LN.** (**a**) *In vivo* comparison of fluorescence intensity of 5K-HA-HPPS in T-MLNs and Inf-LNs at 0.5, 6, and 24 h. Data were presented as mean ± SD, n = 6 mice/group (two-tailed t-test). (**b**) After injection of 5K-HA-HPPS in inf-LNs at 6 h, the Inf-LNs were removed for fixation, dehydration, and labeling F4/80, CD11c, Lyve-1, B220 and CD3 antibodies, confocal imaging was then performed. n = 3 mice.


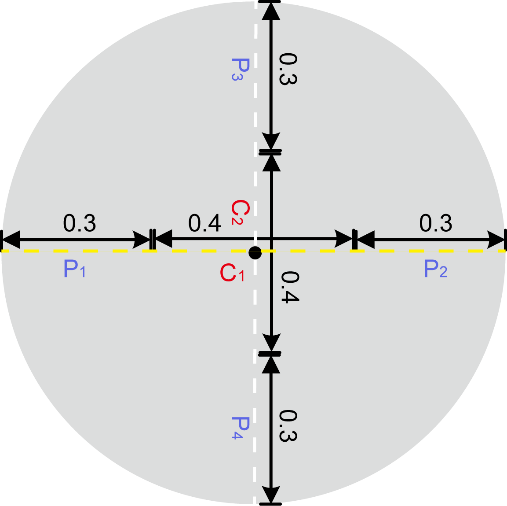


**Fig. S9. Schematic for quantification the PA signal for LNs with different statuses.** Quantification of PA signals along the transverse (yellow line) and longitudinal (white line) diameters of normalized LNs in different statuses. C: Center, P: Periphery.


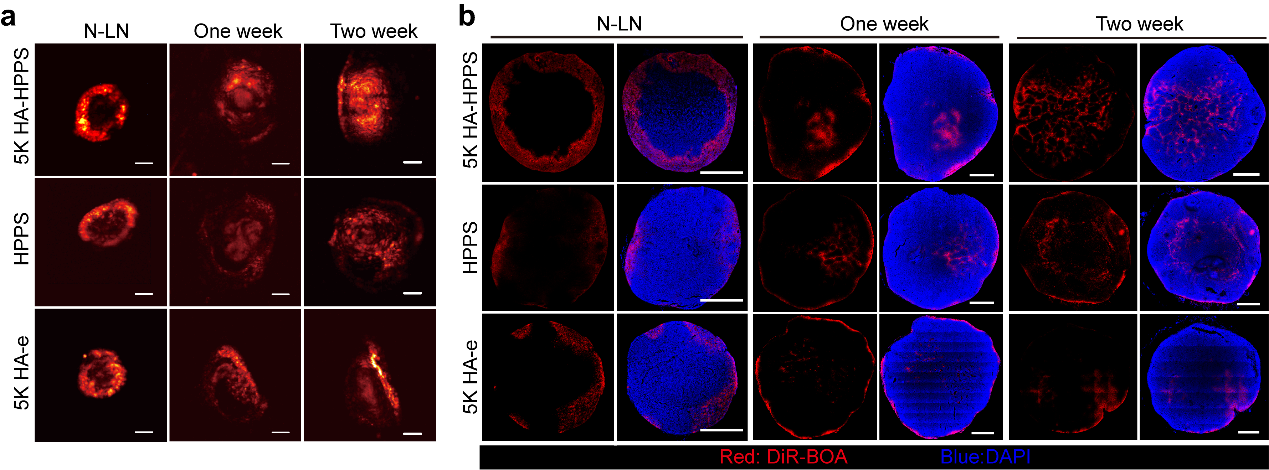


**Fig. S10.** **PAM of SLNs in early tumor metastasis.** (**a**) *In vivo* PAM of 5K-HA-HPPS(DiR-BOA) (upper), HPPS(DiR-BOA) (middle), and 5K-HA-e(DiR-BOA) (down) in N-LNs and early 4T1 metastatic sLNs (1 and 2 weeks) obtained using the AR-PAM system 6 h after intratumor or footpad injection, respectively. Scale bar: 500 μm. n = 3 mice/group. (**b**) Confocal microscopy verified 5K-HA-HPPS(DiR-BOA), HPPS(DiR-BOA), and 5K-HA-e(DiR-BOA) signal distribution in N-LNs and early metastatic SLNs.


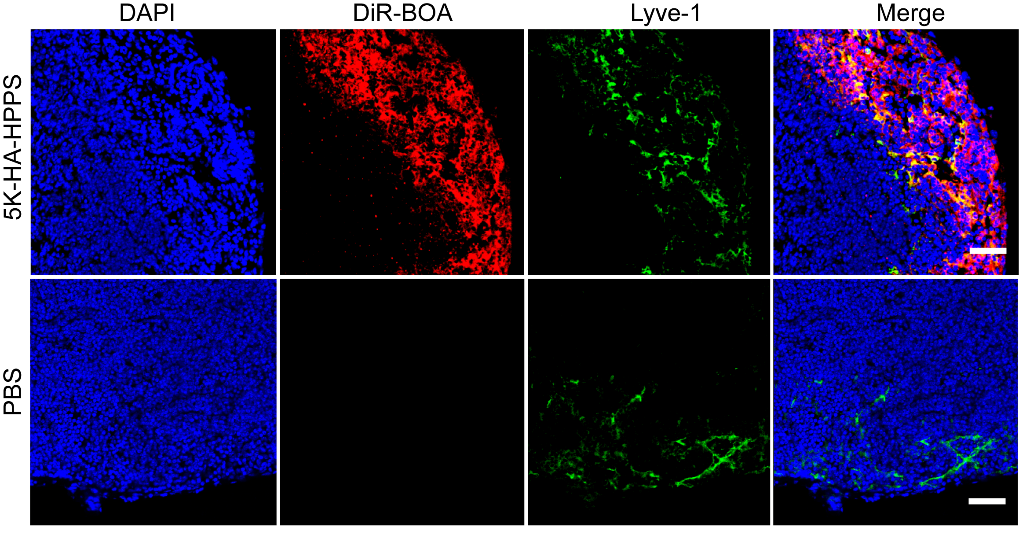


**Fig. S11. Confocal microscopy of 5K-HA-HPPS binding with Lyve-1.** 12 h after injection of 5K-HA-HPPS, frozen sections of N-LNs were taken and labeled with AF488-Lyve-1 antibody. Blue: DAPI, red: DiR-BOA, green: Lyve-1. Slices: 10 μm. Scale bar: 50 μm.
